# Development of a Wearable Camera and AI Algorithm for Medication Behavior Recognition

**DOI:** 10.3390/s21113594

**Published:** 2021-05-21

**Authors:** Hwiwon Lee, Sekyoung Youm

**Affiliations:** 1 InHandPlus, Inc., Seoul 06248, Korea; hlee@inhandplus.com; 2 Department of Industrial and Systems Engineering and Gerontechnology Research Center, Dongguk University, Seoul 40620, Korea

**Keywords:** medication recognition, action recognition, object detection, medication monitoring, smartwatch, wearable device

## Abstract

As many as 40% to 50% of patients do not adhere to long-term medications for managing chronic conditions, such as diabetes or hypertension. Limited opportunity for medication monitoring is a major problem from the perspective of health professionals. The availability of prompt medication error reports can enable health professionals to provide immediate interventions for patients. Furthermore, it can enable clinical researchers to modify experiments easily and predict health levels based on medication compliance. This study proposes a method in which videos of patients taking medications are recorded using a camera image sensor integrated into a wearable device. The collected data are used as a training dataset based on applying the latest convolutional neural network (CNN) technique. As for an artificial intelligence (AI) algorithm to analyze the medication behavior, we constructed an object detection model (Model 1) using the faster region-based CNN technique and a second model that uses the combined feature values to perform action recognition (Model 2). Moreover, 50,000 image data were collected from 89 participants, and labeling was performed on different data categories to train the algorithm. The experimental combination of the object detection model (Model 1) and action recognition model (Model 2) was newly developed, and the accuracy was 92.7%, which is significantly high for medication behavior recognition. This study is expected to enable rapid intervention for providers seeking to treat patients through rapid reporting of drug errors.

## 1. Introduction

Digital innovation technologies, such as the internet of things (IoT), artificial intelligence (AI), mobile devices, big data, and the cloud, are combined with a medical system that has relied on analog technology. Medical information is converted into digital data. Personalized medical services that analyze and use these data have begun to be provided [1]. In particular, the commercialization of 5G technology is expected to develop the digital healthcare market further. In addition, with the use of big data and the development of AI technology, the application possibilities in the field are endless, and the application of digital and AI technology is expected to create a new market in the medical information/system field, where the manual system still dominates [2]. Through this, effective patient health tracking based on technology and wearable devices, reduction of false diagnosis rates by medical staff, and cost savings for medical institutions and pharmaceutical companies have been reported [3,4].

Overall, the industry needs efficient medication management based on technology and data for improved medication compliance [5]. Although the number of people taking medication has increased due to an aging society and increasing elderly population, the tax wasted due to poor adherence to medications is enormous. Approximately KRW (Korean won) 300 trillion in the United States is wasted every year. For pharmaceutical companies, due to low medication compliance, more than 90% of clinical trials are extended, resulting in unnecessary costs. In clinical trials, which cost an average of KRW 45 billion, a 40% improvement in medication compliance can save KRW 14.3 billion. In addition, when an insured individual fails to manage their medication effectively, there are cases in which an insurance fee must be paid when their health deteriorates. Conversely, if the insured remains healthy, the loss ratio of the insurance company decreases. In hospitals, the most basic data for patient care must be identified regarding when and which drugs are taken (medication history data). Depending on how the patient takes the drugs outside the hospital, the drug dose may be changed, or a different prescription may be created, but the problem is that no data currently exist concerning the medication history and that the patient’s memory is subjective [6].

From the standpoint of patients, such as chronically ill and mentally ill patients, many concerns exist about whether patients taking many drugs take the drugs properly and on time. In addition, when several medications are taken, side effects may occur due to poor medication management. Accordingly, a convenient medication management solution is needed for high-quality health management.

Medication nonadherence is extremely common among patients with chronic diseases, such as diabetes or hypertension. Approximately 40% to 50% of them do not adhere to their long-term medications. Medication nonadherence is thought to be responsible for at least 100,000 preventable deaths and USD 100 billion in preventable medical costs per year in the USA [7,8]. However, it remains a pervasive issue within the healthcare sector, especially among older adults, more than 50% of whom live with multiple chronic illnesses that require multiple medications, which reduces adherence. Thus, an appropriate medication management approach that facilitates routine monitoring and assessment of the individual’s adherence is crucial to improving health outcomes and reducing healthcare expenditure [9].

It is difficult to create real-time data from handwritten medication records, and data reliability is problematic because clinical outpatient participants often rely on memory when visiting a hospital. Even if an error is found in the clinical data, it is impossible to verify and trace the existing data due to the limited data verification based on interviews with clinical participants, and unnecessary human resources are wasted. This result leads to an extension of the cost and period due to the additional recruitment of clinical participants.

Medication monitoring implies using some methods to observe whether the patient has taken the medication. Hence, the effectiveness of the monitoring method plays a central role in this regard. Recently, AiCure Inc. developed AI technology-based approaches to optimize patient medication compliance. Similarly, Emocha Mobile Health Inc. developed a video application to observe therapy to enable patients to record themselves taking their pills using their smartphone. However, these methods are not widely used, which may be because, although these methods are user-friendly, they are fully reliant on patients for collecting data.

Therefore, this research aims to develop a novel AI-based medication monitoring system based on a wearable device using deep learning methodology. This research explores the possibility of observing and tracking the medication adherence of patients through an AI-based smart medication monitoring approach using a wearable device. In conducting the study, data were collected from the image sensor of the smartwatch, and an algorithm that can examine the accuracy of the medication-taking behavior was derived using the collected data.

## 2. Related Work

Various technologies have been developed and commercialized for patient medication management. Proteus Digital Health developed eating sensors and abdominal patches, allowing the patient’s medication adherence to be monitored through data-based patient medication management using the solution. Based on its high technology, it is active as a key player in the medication management market, but the disadvantage is that the price is high, and the customer feels considerably resistant when they use a sensor and attach a patch on the neck [10].

AiCure uses a smartphone to photograph a patient’s medication behavior directly and applies technology to determine the presence or absence of medication based on AI technology in clinical trials. According to AiCure, clinical trial costs have been reduced by improving the existing 58% to 62% clinical participant compliance to 92%. However, turning on the mobile phone every time to take a picture is inconvenient, making it difficult to use the service continuously [11]. Smart Pill Bottle is an application that can manage medication. Its advantage is the function of sounding an alarm on a smart medicine box at the time of medication, but the data on when and what medication the user takes are inaccurate. Due to the inaccuracy of the medication data, significant purchases are not made at pharmaceutical companies and hospitals [12].

The earliest studies on object recognition and human recognition technology deployed the convolutional neural network (CNN). However, simple usage of the CNN requires a tremendous amount of training data and time. With the introduction of the object detection technology that classifies objects using bounding boxes, the accuracy of the algorithms used for object detection has significantly increased [13]. The region-based CNN (R-CNN), the basis of object detection, first extracts the region of interest and implements the decision process on the individual algorithm group. The advantage of the R-CNN is that it can extract regions efficiently, even with a few datasets. The R-CNN algorithm was developed in 2013. Subsequently, various object detection algorithms, such as the fast R-CNN, faster R-CNN, You Only Look Once, and RefineDet, have been developed [14].

A 2014 study on object detection performed video image classification using the Sports-1M dataset, which consists of selected YouTube sports videos [15]. The sports videos were sorted into 487 feature labels. Each video image data class was classified using the fusion CNN, which combines two or more spatial and temporal frames into a single frame and analyzes them to achieve video image classification.

A study conducted in 2017 divided the intervals of the objects in the image data, using the intervals to train the CNN model, and finally sent the feature extraction results to the long short-term memory (LSTM) model to achieve object detection [16]. The analysis data were videos of pedestrians from the CUHK Square dataset and the MIT traffic dataset, which is related to vehicle detection. The CNN model and LSTM model, optimized for image analysis and long-term memory, respectively, were used to learn the image features derived from the CNN through the LSTM, where this pattern was remembered in the long term. Object detection was performed by labeling the objects.

A study from 2018 performed detection with enriched semantics where a single image was detected, and the model performance was evaluated using the PASCAL VOC and MS COCO detection data. The detection with enriched semantics model was trained using six consecutive activation maps, where each activation map provided values to the multibox detection. Then, the model integrated six activation maps to detect objects in each image through the trained model [17]. Object detection is applied in various areas, including autonomous driving, CCTV, surveillance, and sporting events. This study employs the fast R-CNN and faster R-CNN frameworks.

Human action recognition, like object detection, is the subject of continuous research. A study conducted in 2015 performed action detection by conducting training on each frame of a person’s movements in video images and dividing this into five parts. Motions were detected by extracting poses in the video clip frame by frame and dividing the motion pattern according to the section of extracted images to recognize the action [18].

Regarding action detection in video clips, a 2017 study extracted the feature values from each image using the CNN model and performed pose estimation by determining the weights of the joints in each image pose. In addition, the weights extracted from the joints were sent to the pooling layer, and the derived values were used to derive the classes of corresponding values using an LSTM model. Then, the recurrent pose-attention network was used to perform human action recognition [19].

In 2019, Hong et al. conducted a recognition study on specific activities by observing human activities concerning the surrounding objects. For the analysis, the Human Interaction with Common Objects dataset was used to recognize a specific activity based on the interaction of humans with the given objects, and the RGB and flow stream values for behavior images were extracted. Then, the pose and pairwise stream values of the objects were extracted using the mask RCNN to conduct a behavior analysis [20].

Various smart sensors are related to healthcare and complement the activities of health professionals in different ways, such as health management and diet control. As such, various medical smart sensors have been the subject of continuous research. Consequently, various sensor data play supporting roles in health management, and many studies have been conducted on analyzing data on human movements or behaviors.

Various studies on sensors related to health have been conducted. For instance, a 2014 study on obesity examined and classified six activities, such as walking and jogging, through a smartphone accelerometer. The classification results acquired for six body types were 98% accurate, and the physical activities were quantified so that the optimal exercise amount for the user could be determined [21].

In a study conducted in 2016, the motion sensor recognized similar motions as a single activity. These motions were sorted into patterns, and a new pattern was generated based on them. Patterning various abstract human activities into several categories of activity and sorting a wide range of data of various behaviors into several patterns of behavior allowed for effective data collection [22].

In 2017, smartphone accelerometers were used to detect three types of human activity: using a smartphone, walking, and running. The CNN was used to measure the magnitude of the acceleration data along the *x*, *y*, and *z* axes to learn and accurately recognize human movement [23].

A 2018 study collected data on individual behavior related to medication adherence using the accelerometer and gyroscope sensor of a smartwatch. Subsequently, classification was performed on the collected data using the random forest algorithm to prevent medication errors [24]. As mentioned above, although many studies have been conducted on behavior classification and extensive sensor data-based research, behavior recognition and sensor data have not been maximized to facilitate intuitive health assistance.

A smartwatch featuring a sensor was implemented in a recent study to develop technology that can identify posture using two or more motion sensors [25]. In addition, an ankle-type internet of things (IoT) device has been developed for predicting various chronic diseases, such as Alzheimer’s disease [26].

The current study has also developed a human behavior recognition method. This method is for detecting medication-taking activity using smartwatch sensors and the AI algorithm.

## 3. Materials and Methods

**Figure 1:**
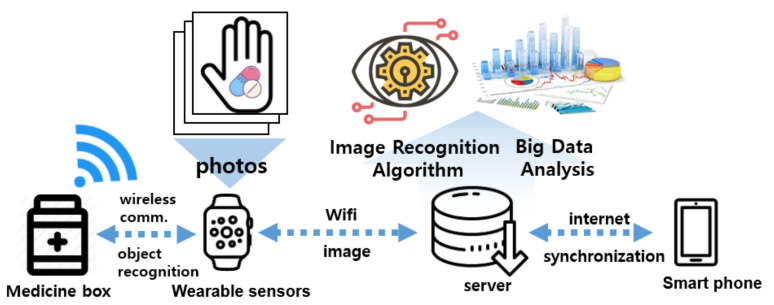
Overview of the medication monitoring system using a wearable device**.**

The smartwatch-based automatic medication behavior monitoring system developed by InHandPlus, Inc. (Seoul, Korea) is designed to enable not only automatic medication behavior analysis but also customized healthcare services based on the user’s medication history data by applying AI technology. Figure 1 presents an overview of the medication monitoring system using a wearable device.

**Figure 2:**
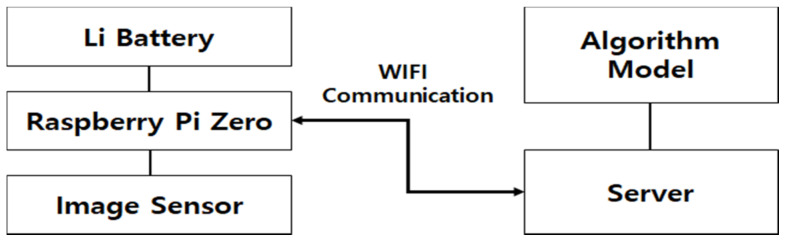
Structural diagram of a smartwatch implemented with Raspberry Pi**.**

Before the final product was completed, a prototype was developed to collect data. First, using a wearable device that includes a camera module based on Raspberry Pi (Raspberry Pi Foundation, Cambridge, UK), scenes taken by the patient when taking medicine were collected, and a battery and power switch were added for portability. The configuration diagram of the smartwatch designed accordingly comprises a Raspberry Pi, lithium-ion battery, and image sensor in Figure 2.

**Figure 3:**
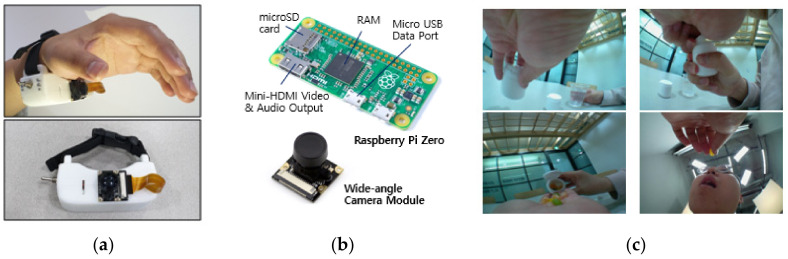
(**a**) Wearable device for monitoring medication behavior with a camera module applied. (**b**) Configuration of the internal module of the wearable device. (**c**) Example of medication behavior data taken from the wearable device**.**

Figure 3 illustrates an example of a wearable device with an applied camera module for monitoring medication behavior. The figure also presents the internal module configuration of the wearable device and the medication behavior data taken from the wearable device.

For the main controller, Raspberry Pi Zero was used. The main reason for using Raspberry Pi was that the module control, Wi-Fi, and Bluetooth interworking were simple. This system works in a general Wi-Fi environment, and it takes around 2 s to upload a video of around 20 s in length. Moreover, since the video is sent sequentially, there is no difficulty in Wi-Fi traffic. If traffic occurs in the server due to a large number of users at the same time, it is temporarily stored in the smartwatch memory, and video data are sequentially sent from the watch to the server when the traffic is reduced. The camera was controlled using the Pi-camera module of Raspberry Pi Zero, and the HTTP web protocol was used for the file transfer.

For the image sensor, the researcher experimented by attaching image sensors to various locations, and when a camera lens was placed on the lower part of the hand, pills could be easily recognized while taking medication. Accordingly, the prototype was configured to attach the camera to the bottom of the wrist. The component used as the image sensor was the Omnivision 5647 Sensor (ArduCam, https://www.arducam.com/ (accessed on 21 May 2021); wide-angle camera module), an image sensor for Raspberry Pi.

For the battery, a lithium-ion battery with a capacity of 300 mA was used so that it could be fully employed for research without any trouble when wearing it. The six hours are the duration of the battery used when the camera is running. The camera works only when taking medicine, and assuming that one person takes medicine three times a day, the battery lasts more than 24 h. Its size was 6 cm × 1.2 cm × 3.5 cm. In this case, the maximum recording time was around 6 h.

**Figure 4:**
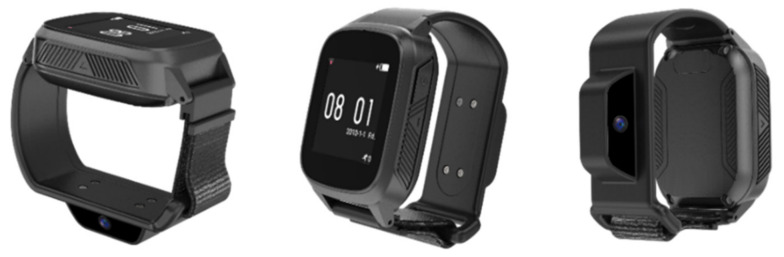
Camera module embedded in smartwatch for medication behavior monitoring.

**Figure 5:**
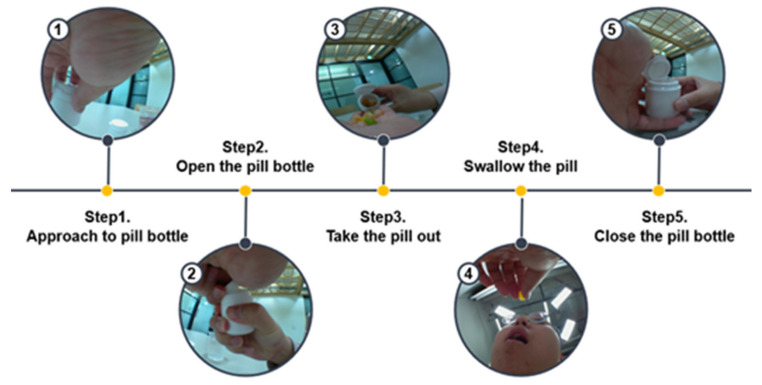
Example steps of the medication behavior recorded by the smartwatch.

The final product includes a small camera module embedded on the smartwatch band developed by InHandPlus, Inc. (Figure 4). This smartwatch can monitor the user’s hand motions and objects grabbed by the hands. This smartwatch was introduced in this study to monitor pill-taking behavior and propose a new method for a medication management solution based on real-world evidence: medication-taking history data. In addition, to analyze medication behavior, more than nine specific features and steps, including “opening the pill bottle” and “swallowing the pill,” are deduced (Figure 5). The collected video data monitored by the smartwatch were transferred to the computer server for further analysis by the AI algorithm, which InHandPlus also developed.

### 3.1. For Object Detection (Module 1)

A model using the regions with convolutional neuron networks (R-CNN) learning model to detect objects and the CNN and support vector machine (SVM) models to perform medication recognition was derived to analyze the medication-taking activity. It contains the meaning of a neural network that performs object detection by using the set region as a feature (input value) of the CNN. R-CNN is the first model to apply CNN to the object detection field, and it is a meaningful model showing that the detection method using CNN can lead to a high level of performance not only in the classification but also in the object detection field.

The basic structure of R-CNN can divide the entire task into two steps, as it is called a 2-stage detector. First, it uses region proposal to find the location of an object and region classification to classify the object. The structure of R-CNN, which is performed to handle these two tasks, can be described with a total of four modules: (1) region proposal to find the area of the object regardless of the category after inputting the data and label in the image; (2) warping/crop a feature vector of a fixed size from the proposed region and use it as an input of the CNN; here, CNN uses a network that has already been pretrained using ImageNet; (3) classification through support vector machine (SVM), which is a linear supervised learning model, using feature maps from CNN, and (4) bounding box regression through regressor.

### 3.2. For Action Recognition (Module 2)

The second model that performed classification applied the Long Short-Term Memory models (LSTM) to the weighted data stream of the results obtained from Model 1 and performed a classification based on the size and type of the detected object. The LSTM is known as an algorithm suitable for predicting continuous time series events. It is advantageous in dealing with sequence data and is known to be suitable for problems with temporal ordering. It has long-term dependencies that can remember old information. LSTMs also have the chain-like structure, but the repeating module has a different structure: instead of having a single neural network layer, there are four, interacting in a very special way. Equation (1) represents LSTM.

*f_t_* = *σ*(*W_xh_f_*·*x_t_* + *W_hh_f_*·*h_t−1_* + *b_h_f_*) *i_t_* = *σ*(*W_xh_i_*·*x_t_* + *W_hh_i_*·*h_t−1_* + *b_h_i_*) *o_t_* = *σ*(*W_xh_o_*·*x_t_* + *W_hh_o_*·*h_t−1_* + *b_h_o_*) *g_t_* = tanh(*W_xh_g_*·*x_t_* + *W_hh_g_*·*h_t−1_* + *b_h_g_*) *c_t_* = *f_t_* ⊙*c_t−1_* + *i_t_* ⊙*g_t_* *h_t_* = *o_t_* ⊙tanh(*c_t_*) (1)

The forget gate *f_t_* is a gate for ‘forgetting past information’. The value sent by the forget gate is the value obtained by taking the sigmoid after receiving *h_t−1_* and *x_t_*. Since the output range of the sigmoid function is between 0 and 1, if the value is 0, the information of the previous state is forgotten, and if it is 1, the information of the previous state is completely memorized. Input gate *i_t_*⊙*g_t_* is a gate for ‘memorizing the current information’. It receives *h_t−1_* and *x_t_*, takes a sigmoid, and takes the hyperbolic tangent with the same input, and then the Hadamard product operation is the value that the input gate sends. Individually, *i_t_* ranges from 0 to 1 and *g_t_* ranges from −1 to 1, so they represent the intensity and direction, respectively.

**Figure 6:**
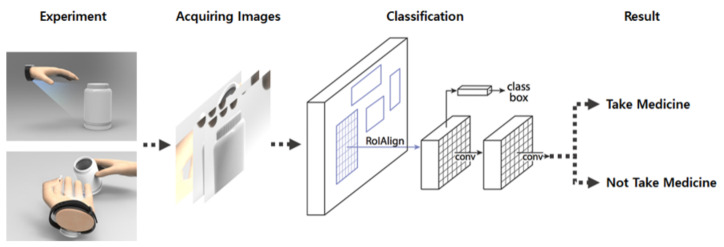
Experimental model for medication behavior recognition.

Figure 6 describes the overall process. In the result stage, if it is confirmed that a pill is put in the mouth in a series of shots, it is recognized that the pill has been taken, and if there is only a scene holding the pill in the hand, it is recognized as not taking the pill. By first extracting the various weights of the objects through the R-CNN model, it became possible to achieve highly accurate results using fewer data, unlike the basic CNN model.

## 4. Experiments

The experiment was conducted on 89 volunteers between the ages of 20 and 60 (22 males with an average age of 38.2 and 67 females with an average age of 33.3). The experimental group consisted of the general population who commonly take medication. As for the experiment method, each subject was given a cup of water and a plastic bottle containing pills. The experiment was conducted by filming them for approximately 15 to 20 s while taking the medication. The ethics committee of Dongguk University approved the testing (DUIRB-201908-02).

The participants wore the smartwatch and were asked to take their medication ordinarily, without paying attention to the smartwatch, to simulate a real-life medication-taking situation as closely as possible. In addition, the participants were requested to take the medication at their usual speed, without delays due to being conscious of filming.

Considering that the average length of the medication-taking activity was approximately 15 to 20 s, the average dataset contained 250 images when the clips were converted to frame-by-frame images. Approximately 200 video clips were taken. They were all converted into separate frame images, totaling 50,000 image sets.

After data collection, the training process was conducted using the collected data. Of the 50,000 images collected for the architecture for Model 1, 40,000 (80%) were used as training data, and 10,000 (20%) were used as testing data.

For each image, medication-taking-related objects, such as the face, cup, pill, and pill bottle, were labeled. The criteria for selecting each object were based on noticeable features in taking medication that should be closely monitored by someone monitoring the medication-taking process.

Model 2 consisted of 270 datasets: 160 for the training set and 110 for the testing set. Each dataset consisted of 50% medication-taking activity and 50% non-medication-taking activity.

**Figure 7:**
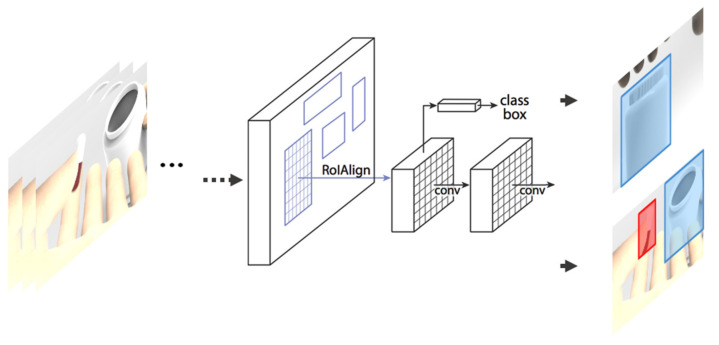
R-CNN classification model for object detection.

**Figure 8:**
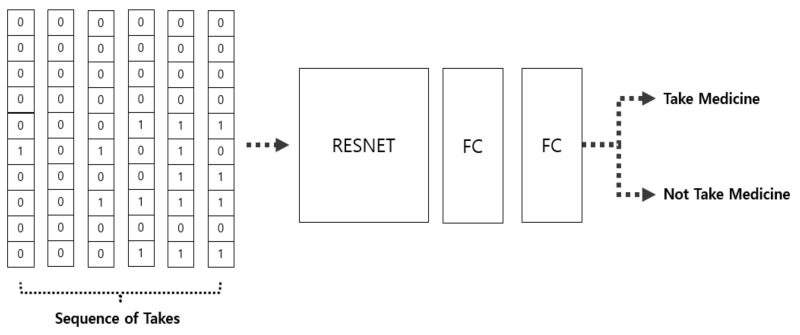
CNN classification model for medication behavior recognition**.**

Figure 7 shows the R-CNN classification model for object detection. Figure 8 shows the process of how to determine whether or not to take medication by using the data extracted through object detection during the “classification” in Figure 7. Sequence of takes is object information extracted from each video. The extracted information is input to the artificial intelligence model and returns the presence or absence of medication.

Inception-V3 was used for object detection, and the existing trained model was fine-tuned [27]. A TensorFlow graphics processing unit was run using multiple GPUs. Xeon processor (Intel, Santa Clara, CA, USA) and Nvidia GeForce 1080Ti graphic cards (Nvidia, Santa Clara, CA, USA) were used to implement the training model. TensorFlow and Keras libraries were used.

## 5. Results and Discussion

**Figure 9:**
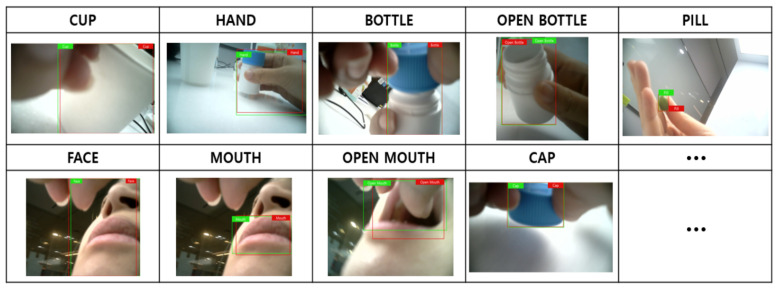
Object detection examples for Model 1.

The experimental results revealed that Model 1 recognized each object very accurately, as indicated in Figure 9. Overall, most objects were detected effectively, and the mean average in the 0.5 intersection over union was 86.1%.

Table 1 shows the results for object recognition. The experiment in Phase 2 was determined based on the results with or without medication from Model 1. The algorithms in the experiments were the basic CNN, logistic regression, boosted decision tree, SVM, and decision forest. Logistic regression is a statistical model that, in its basic form, uses a logistic function to model a binary dependent variable, although many more complex extensions exist. Boosting in a decision tree ensemble tends to improve accuracy, with some small risk of less coverage. The support vector machines (SVMs) are supervised learning models with associated learning algorithms that analyze data for classification and regression analysis. A decision tree is a predictive model that recursively partitions the covariate space into subspaces such that each subspace constitutes a basis for a different prediction function.

From the experimental results in Table 2, our model had the highest accuracy at 92.7%. The logistic regression, boosted decision tree, SVM, and decision forest algorithms were also evaluated, but they all exhibited lower accuracy than our CNN model. The actual accuracy check results using the confusion matrix revealed that the proposed method exhibited a high overall accuracy, with three false-positive cases and five false-negative cases out of 110 total cases as shown in Table 3.

This study derived an AI algorithm that can perform medication behavior recognition through a camera sensor embedded in a smartwatch developed by InHandPlus, Inc. The algorithm was applied to 110 test datasets, and the results were 92.7% accurate. The high accuracy appears to be primarily attributable to the ability of the R-CNN (Model 1) to find features very accurately, which facilitates accurate object classification during the second classification phase (Model 2). The accurate identification of human medication-taking action using continuous camera data could contribute to expanding the range of applications to other human behaviors, such as inhaler usage, glucometer usage, autoinjector usage, and eating behavior, in the future.

The existing studies on action recognition can be classified into two main categories: patient posture recognition based on a motion sensor, such as a gyro sensor [28,29], and action recognition using static data captured by a third person [18,19,30,31]. The first method has the advantage of requiring low power and high wearability. However, its low accuracy is a major shortcoming. In contrast, action recognition algorithms have limited applications and can only be implemented in certain areas, such as security cameras and automotive dash cameras. In this study, instead of using these rather limited methods, we implemented the camera sensor in the smartwatch to derive an algorithm that can detect patient behavioral patterns related to medication adherence. We obtained satisfying results in terms of both accuracy and usability. The AI algorithm that performs gesture recognition through the camera data can be employed in various areas in the future, such as medication management services, to detect patient medication adherence and allow health professionals to respond appropriately.

**Figure 10:**
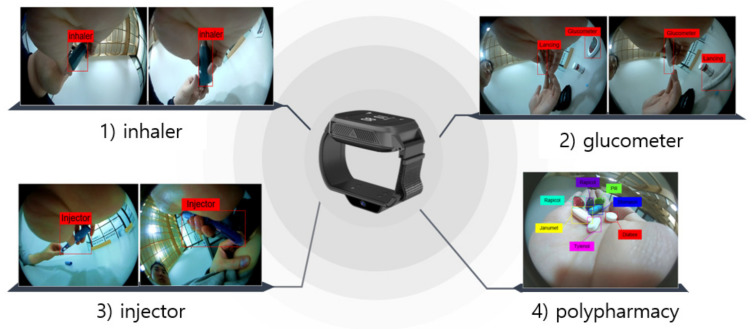
Expansion of smartwatch-based AI analysis targets.

In addition to this study, we experimented with the possibility that the smartwatch-based AI behavior analysis technology is not limited to taking one type of pill, and it is possible to analyze any behavior or object held in the hand. As presented in Figure 10, the technology could be applied to patients with diabetes or asthma, and a recognition rate similar to that for the pills could be derived. Examples include glucometers used daily by patients with diabetes, inhalers used by patients with asthma with respiratory symptoms, expensive autoinjectors used by patients with cancer, and taking several types of pills simultaneously. The technology can be extended to analyzing multiple medicinal products for patients. In addition, it is possible to expand specific food and beverage analyses for obesity, high blood pressure, and diet.

## 6. Conclusions

A digital medication management platform was developed by applying a smartwatch and AI technology for medication management to solve the problem of noncompliance with medication, which is a social issue. In addition, the intention was to provide personalized healthcare services and insight on using medication data.

The system proposed in this study enables the application of customized healthcare services based on the user’s medication history data and automatic medication behavior by applying AI technology. This system is an image-based medication behavior analysis system using an AI method to improve analysis accuracy and learning speed. It is an automated system that automatically collects data while wearing a smartwatch and completes analysis based on the AI. It is a technology that does not require any behavior change if the user wears only the watch.

This study developed a new concept and method for medication behavior recognition using a camera-embedded smartwatch and AI algorithm. We proposed a method of assessing the medication state of a patient taking medication by mounting a camera on a wearable device with an image sensor and learning the captured data by applying the latest CNN. This technique provides image-based classification so that the patient’s medication status can be accurately recognized.

This method uses a novel technique of collecting image data for the AI application, focusing on hand movements monitored by the smartwatch. The high accuracy of medication behavior recognition of over 92% demonstrates the potential of this method to apply to medication management for chronic patients.

Through rapid reporting of medication errors, providers who want to treat patients can provide quick interventions. Clinical researchers can change experiments and predict various health levels through medication compliance. However, there is a limitation that the test was conducted in a laboratory environment. Tests in various environments will be supplemented to confirm robustness. Future studies are expected to improve the accuracy of the proposed method by collecting more data from various situations. In addition, the usability of the proposed method can be extended by considering the variety in the objects of interest, such as different types of pills, syringes, glucose meters, and medical inhalers, while performing behavior analysis. Currently, the three types of pills are only classified automatically, but by learning various pills, it is expected that even the types of pills can be distinguished. In addition, a smart alarm function based on an algorithm recognizing the user’s arm pattern using a motion sensor will be developed to enable recognition of medication and eating behavior using a smartwatch. We want to implement an alarm function to promote taking medication according to the actual meal time through repetitive learning of the Pak pattern, which is expected to reduce human intervention further and increase medication compliance.

This system can be applied to medication management and a digital clinical platform for clinical trial participants in the future. It is expected to be possible to apply a non-face-to-face telemedicine platform for preventing and managing infectious diseases, such as coronavirus disease 2019 (COVID-19).

## Figures and Tables

**Table 1:** Results of AP@0.5 on object examples.

Category	AP@0.5 IOU
Bottle	0.939137
Cap	0.89423
Cup	0.831954
Face	0.954092
Hand	0.857066
Mouth	0.777187
Open bottle	0.83886
Open mouth	0.947501
Pill	0.827504
Average	0.87417

**Table 2:** Medication recognition algorithm accuracy.

Method	Accuracy	Precision	Recall
Logistic regression	0.845	0.81	0.887
Boosted decision tree	0.882	0.9	0.849
Support vector machine	0.909	0.939	0.868
Decision forest	0.918	0.923	0.906
Our Model (CNN)	0.927	0.909	0.949

**Table 3:** Confusion matrix regarding medication film results.

	Medication-Taking Activity	Non-Medication-Taking Activity
Medication-taking activity	50	5
Non-medication-taking activity	3	52

## Data Availability

The data presented in this study are available on request from the corresponding author. The data are not publicly available due to proprietary information.
